# Coccidioidomycosis Skin Testing in a Commercially Insured Population, United States, 2014–2017^1^

**DOI:** 10.3201/eid2603.190798

**Published:** 2020-03

**Authors:** Kaitlin Benedict, Orion Z. McCotter, Brendan R. Jackson

**Affiliations:** Centers for Disease Control and Prevention, Atlanta, Georgia, USA

**Keywords:** coccidioidomycosis, skin tests, United States, health insurance, respiratory infections, Coccidioides

## Abstract

Coccidioidomycosis skin testing appears to be uncommon, based on US health insurance claims data. Patient demographic features were consistent with the approval of the test for adults, but few patients had previous coccidioidomycosis diagnosis codes supporting its use for detecting delayed-type hypersensitivity in those with a history of pulmonary coccidioidomycosis.

Coccidioidal skin testing has been a valuable epidemiologic and clinical tool for estimating the prevalence of previous *Coccidioides* spp. exposure and monitoring treatment response (*1*–*3*). Such testing could also be useful for evaluating healthy persons’ risk of developing coccidioidomycosis (*3*). The skin test became commercially available again in 2014 after more than a decade; it is approved for adults 18–64 of age who have a history of pulmonary coccidioidomycosis (*3*,*4*). However, little is known about its use in the general population with unknown exposure to *Coccidioides*. We describe features of patients who have employer-sponsored insurance who received a *Coccidioides* skin test.

We used the IBM MarketScan Research Databases (https://www.ibm.com/products/marketscan-research-databases) to identify patients with a Current Procedural Terminology (CPT; https://www.ama-assn.org/amaone/cpt-current-procedural-terminology) code for a coccidioidomycosis skin test during 2014–2017. MarketScan health insurance claims data include outpatient visits and prescriptions and hospitalizations for employees, dependents, and retirees, representing >25% of all employer-sponsored beneficiaries throughout the United States. This analysis was not subject to review by the Centers for Disease Control and Prevention institutional review board because the data are fully deidentified.

We accessed the data through MarketScan Treatment Pathways, a web-based platform that includes data from persons with health insurance plans that contribute prescription drug data to MarketScan. We limited the analysis to patients continuously enrolled during the 3 months before and after the skin test. We examined periods up to 3 years before and 1 year after; because the primary features of interest did not change substantially, we focused on the smaller period to retain a larger study population.

We analyzed patient demographics; visits within 3 days to estimate the proportion who returned to have their test results read after 48 hours (compared with patients with a CPT code for tuberculosis skin testing); coccidioidomycosis diagnoses (International Classification of Diseases [ICD], 9th Revision, Clinical Modification, codes 115.00–115.99; ICD, 10th Revision, Clinical Modification, code B38); laboratory testing; and fluconazole prescriptions. We also examined certain underlying medical conditions and assessed the cost of skin test claims to patients and insurers among patients with noncapitated health plans.

Among ≈57 million MarketScan enrollees, 505 had a coccidioidomycosis skin test; 407 of those were continuously enrolled. Of those 407, most (n = 391, 89%) were 18–64 years of age, female (n = 243, 60%), and in California (n = 367, 90%) (Table). Thirty-five percent had a code for a subsequent visit within 3 days, compared with 24% of 1,061,118 patients who had a tuberculosis skin test. Test results were not available.

**Table:** Characteristics of patients who received a coccidioidomycosis skin test, 2014–2017, USA

Characteristic	Value	Diagnosis or procedure codes
Age, median, y (range)	46 (2–85)	
0–17	20 (5)	
18–34	73 (18)	
35–44	67 (16)	
45–54	105 (26)	
55–64	116 (29)	
>65	26 (6)	
Sex		
M	164 (40)	
F	243 (60)	
Primary beneficiary’s residence		
California	367 (90)	
Arizona	16 (4)	
Other or unknown state	24 (6)	
Underlying conditions		
Immune-mediated inflammatory disease	44 (11)	ICD-9-CM codes 555, 556, 696.0, 696.1, 696.8, 714.0, 714.2; ICD-10-CM codes K50, K51, L40, M023, M05, M06, M08, M33, M352, M45
Chronic obstructive pulmonary disease	40 (10)	ICD-9-CM codes 490–492, 494, 496; ICD-10-CM codes J41–J44
Diabetes	39 (10)	ICD-9-CM codes 249–250; ICD-10 codes E08–E11
HIV/AIDS	5 (1)	ICD-9-CM code 042; ICD-10-CM code B20
Solid organ or stem cell transplant	4 (1)	ICD-9-CM codes V42 (excluding V42.3–V42.5), 996.8; ICD-10-CM codes T86, Z94 (excluding Z94.5– Z94.7)
Hematologic malignancy	4 (1)	ICD-9-CM codes 200–208; ICD-10-CM codes C81–C86, C88, C90–C96
Fungal laboratory testing in the 3 mo before skin test		
Coccidioidomycosis serologic test	20 (5)	CPT codes 86331, 86171, 86635
Fungal culture	5 (1)	CPT codes 87101, 87102, 87103, 87106, 87107
Fungal smear	11 (3)	CPT codes 87205, 87206, 87210
Fungal laboratory testing on the day of or in the 3 mo after skin test		
Coccidioidomycosis serologic test	63 (15)	CPT codes 86331, 86171, 86635
Fungal culture	34 (8)	CPT codes 87101, 87102, 87103, 87106, 87107
Fungal smear	42 (10)	CPT codes 87205, 87206, 87210

*Values are no. (%) except as indicated. CPT, Current Procedural Terminology; ICD-9-CM, International Classification of Diseases, 9th Revision, Clinical Modification; ICD-10-CM, International Classification of Diseases, 10th Revision, Clinical Modification.

In the 3 months before the skin test, 5% had a coccidioidomycosis diagnosis code, 5% had a coccidioidomycosis serologic test code, and 5% had a fluconazole prescription. On the skin test date and in the 3 months after, 7% had a coccidioidomycosis diagnosis code, 15% had a serologic test, and 9% had a fluconazole prescription. Forty-four patients (11%) had noncapitated health plans; among those, the mean cost of skin test claims was $43.66 (range $0–$264). Mean costs were $31.57 (range $0–$184) to insurers and $12.09 (range $0–$264) to patients.

In the context of the large at-risk population in *Coccidioides*-endemic areas, coccidioidomycosis skin testing appears to be uncommon in this privately insured population. Real-world data on the test’s use and performance in the general population are lacking, although it performs well for risk-stratifying prison inmates (*5*). Reasons for its low use could be its limited approved clinical indication to detect delayed-type hypersensitivity to *Coccidioides* in persons with a known history of disease or that the clinical implications of such testing may be unclear. Cost may also play a role, although it is unclear why most patients had capitated health plans. Reasons why most tests were performed in California rather than in Arizona (states where most coccidioidomycosis cases occur) are unknown.

Patient age was consistent with the test’s approval for use in adults. However, few patients had coccidioidomycosis diagnosis codes, suggesting possible use of this test to screen for immunity in those with unknown exposure to *Coccidioides*, which has not been evaluated. Another explanation for the low frequency of coccidioidomycosis diagnosis codes in the 3 months before testing is a more distant coccidioidomycosis history. We observed laboratory testing and fluconazole prescription patterns that suggest that the test might be occasionally used as a supplemental diagnostic tool.

Patient return visit rate (35%) was comparable to that of tuberculosis skin testing. This proportion could appear falsely low if providers chose not to bill for reading the test results. In addition to lack of test results, limitations of this analysis include potential coding misclassification.

In summary, skin testing could be useful for evaluating healthy persons’ risk of developing coccidioidomycosis but appears to be rare, even in endemic areas. Determining features of patients who receive a coccidioidomycosis skin test and assessing clinicians’ knowledge and attitudes could provide insight into the test’s clinical and epidemiologic value.
